# Information Conversion in Measuring Channels with Optoelectronic Sensors

**DOI:** 10.3390/s22010271

**Published:** 2021-12-30

**Authors:** Vasyl V. Kukharchuk, Sergii V. Pavlov, Volodymyr S. Holodiuk, Valery E. Kryvonosov, Krzysztof Skorupski, Assel Mussabekova, Gaini Karnakova

**Affiliations:** 1 Faculty for Power Engineering and Electromechanics, Vinnytsia National Technical University, Khmelnytske Shose 95, 21021 Vinnytsia, Ukraine; bkuch@ukr.net (V.V.K.); vgolodyk@gmail.com (V.S.H.); 2 Laboratory of Biomedical Optics, Faculty for Infocommunications, Radioelectronics and Nanosystems, Vinnytsia National Technical University, Khmelnytske Shose 95, 21021 Vinnytsia, Ukraine; psv@vntu.edu.ua; 3 Department of “Engineering and Technology” of the Azov Maritime Institute, NU “Odessa Maritime Academy”, st. Chernomorskaya, 19, 87517 Mariupol, Ukraine; Yhtverf007@ukr.net; 4 Faculty of Electrical Engineering and Computer Science, Lublin University of Technology, Nadbystrzycka 38d, 20-618 Lublin, Poland; 5 Academy of Logistics and Transport, 97 Shevchenko st., Almaty 050012, Kazakhstan; asel_1989_09@mail.ru; 6 M.Kh.Dulaty Taraz Regional University, Tole Bi St 40, Taraz 080000, Kazakhstan; gaini.karnakova@mail.ru

**Keywords:** conversion of measuring information, optoelectronic sensor, measuring transducer, analog-to-digital converter, quantization, conversion function, sensitivity equation

## Abstract

The purpose of this work is the authors’ attempt to identify the main phases of information transformation in measurement channels on the example of an optical measurement channel with microprocessor control. The authors include such phases: hardware implementation and analytical representation of an optical sensor’s converting functions and a current-to-voltage converter; based on the methods of experimental computer science, the converting functions and sensitivity are deduced, analytical dependences for estimation of a range of measurement are obtained. It is shown that the choice of information transmission type in the microprocessor measuring channel significantly affects the speed of the measuring channel. Based on the uncertainty in the form of entropy before and after measurements, the amount of information for measuring channels with optoelectronic sensors is estimated. The application of the results obtained in the work allows even at the design stage of physical and mathematical modeling to assess the basic static metrological characteristics of measuring channels, aimed at reducing the stage of development and debugging of hardware and software and standardization of their metrological characteristics.

## 1. Introduction

Nowadays, there exists is an intensive development of registration, processing, and information storage instruments. As information becomes a strategic resource of society, its storage requires constant improvement of sensory measuring transducers, which ultimately boils down to increasing the productivity of the information measuring systems [1,2,3,4]. One of the broad classes of sensor transducers are optical sensors, where the information about the object of study is provided by converting optical radiation into an electrical signal, and their principle of operation is based on the laws of optics. Prospects for the use of optical sensors are great since light allows us to transform quite large streams of information at low power and without significantly affecting the state of the object, without damaging it, and can penetrate the object to a considerable depth.

Optical methods have several advantages over electrochemical and mechanical methods. The most widely used optical methods are absorption, fluorescence, spectrophotometry, chemiluminescence, photoplethysmography, and surface plasmon resonance [2,5,6,7].

Their main advantages are high speed, measurement accuracy, high sensitivity, wide measuring range, and various applications. The disadvantage is the high cost of measuring channels of conversion and processing [1,7,8,9].

According to the principle of optical–electrical conversion, optical sensors can be divided into four types: those, based on the effects of photoelectron emission, photoconductivity, photovoltaic and pyroelectric.

The class of optical sensors also includes sensors in which the active light is applied according to the laws of reflection and refraction of light, its passage through a heterogeneous medium, the phenomenon of light diffraction, etc., are used to obtain information.

The main advantages over other types of sensors are the possibility of non-contact measurement, the ability to measure the parameters of objects of both extremely large and extremely small sizes, high speed, application of integrated technology that provides small size and long service life, as well as a variety of applications that allow to measure different physical quantities, determine shapes and recognize objects, etc. Along with the advantages, optical sensors have some disadvantages, namely sensitivity to pollution, exposure to extraneous light, light background, as well as temperature, and have a significant cost.

At present, optical sensors are widely used in biology and medicine. However, until recently, as a rule, optical sensors analyzed preparations of biological substances (in vitro). But nowadays, optical sensors allow receiving information from a living object (in vivo), without destroying living tissue, non-invasively, with the least impact on natural processes occurring in humans, animals, and plants. And in these cases, the most valuable research tool is light radiation. Optoelectronic sensors provide only the conversion of the input physical quantity into the output electrical. To obtain the result it is necessary to compare the electrical value of the output of the sensor with the sample. As a result of quantization, a binary code is obtained, which is exported to the microprocessor battery, stored in RAM, and the result with a unit of measurement is obtained from the conversion equation. This whole sequence of measuring information transformation takes place in the measuring channel.

**Figure 1:**
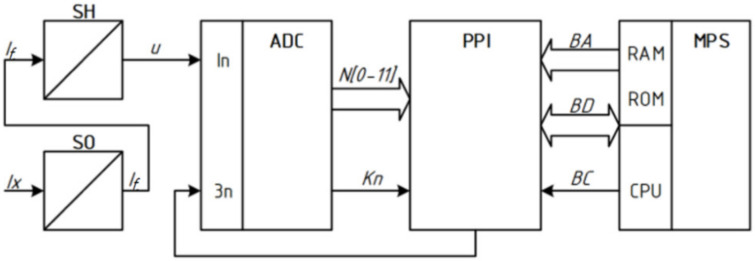
Generalized block diagram of the measuring channel.

The measuring channel (Figure 1) is a set of measuring devices SO and SH, measuring instruments ADC and PPI communication, designed to create measuring information about one physical quantity [3,10,11,12,13,14,15]. The generalized architecture of construction of the measuring channel with the optoelectronic sensor is given in Figure 1.

In this structure, relating to direct conversion circuits, the main components are the following: SO—Optoelectronic sensor; SH—Shunt (current-to-voltage converter; ADC—Analog-to-digital converter; PPI—Parallel programmable interface; MPS—microprocessor system consisting of CPU microprocessor, RAM, and permanent ROM memory; BA, BD, BC—Address bus, data, and management, respectively.

This block diagram is well-known and widely used by the developers of information and measurement technologies. Mathematical models of analog, analog-to-digital, and digital converters, which adequately describe the different phases of information transformation in optical measurement channels, remain unexplored here. The lack of such theoretical results is a scientific and applied problem, the solution of which will allow at the stage of hardware design and development to model such basic static metrological characteristics of the optical channel selected for research as the transformation equation (calibration characteristic), sensitivity, quantization error, measuring range, speed, which together will give new knowledge in the field of metrological support of optical measuring channels.

The aim and scientific novelty of the work is to develop new mathematical models, which are the transformation function of the optical measuring channel, the equation of sensitivity and quantization error, analytical dependences for estimating the measuring range in the form of lower and upper measurement limits, speed.

## 2. Phases of Transformation of Measuring Information in the Optoelectronic Sensor

The input (measured) physical non-electric quantity here is the optical intensity $I_{X}$, which in the process of transformations is converted into a numerical value—the measurements’ result. First, the sensor SO converts the optical intensity into the value of the current $I_{\Phi }$. Next, the physical value of the current $I_{\Phi }$ in the shunt SH is converted into a voltage U, which value is fed to the analog input $I_{n}$ of the analog-to-digital converter ADC, where the signal triggers Зn quantized by size and in the process is converted into 12-bit binary code N [00-11]. Simultaneously with the fixing of the binary code on the digital outputs of the ADC, a signal of logic “1” is formed at the output of the CP (end of the conversion). The unit level of this signal binary code N [00-11] from the digital outputs of the ADC is stored in the input ports PA and PB parallel program interface PPI with simultaneous setting in the port PC [02]: = “1”. The presence of the PC [02]: = “1” flag is perceived by the software driver as a command that records binary code N [00-11] in the input ports PA and PB, can be transmitted via the PPI interface to the accumulator of the microprocessor system MPS, with storing this binary code in the RAM of the microprocessor system MPS [16,17,18].

In order to further develop the applied “cybernetic” science of measurement, we consider in detail the sequence of converting information in the measurement channel and analyze it from the standpoint of mathematics, experimental computer science, and metrology [19,20,21]. Starting with the analysis of the measurement transformation of the optical sensor SO, which means the operation of converting the input non-electric value of the optical intensity $I_{X}$ into the output electric value of the photocurrent $I_{f}$, functionally related dependence [4]:

(1) $$I_{f}=f\left(I_{X}\right).$$

The photocurrent $I_{f}$ at the output of the photodetector is directly proportional to the intensity of the reflected scattered radiation $I_{X}$, for example from a bio-object, is determined [5,6,22,23] as:

(2) $$I_{f}=-S_{I\lambda }\cdot \tau_{\lambda }\cdot I\cdot 10^{-3}\cdot \frac{\tau_{oпт}\cdot \pi \cdot r^{2}}{l^{2}\cdot L^{2}}\cdot \cos\alpha \cdot \cos\beta \cdot W,$$

where $S_{I_{\lambda }}$ is the integral sensitivity of the photodetector (selected from the table with the characteristics of photosensors [6,7] $S_{I_{\lambda }}$ = 0.02; $\tau_{\lambda }$—is the transmittance of the polarized filter in the transducer (0.5 ÷ 0.6); *I*—is light power of the radiation source $I=\frac{P}{S}=5\left[\frac{\mathrm{Wt}}{{\mathrm{cm}}^{2}}\right]$; $\tau_{oпт.}$—is the coefficient that characterizes the passage of light through the optical system (0.9–0.95); *l*—is the distance from the source of radiation to the bio-object $l=\left(2÷3\right)\cdot 10^{-1}\;\mathrm{cm}$; L—is the distance from the bio-object to the photosensor $L=2\cdot l=2\cdot \left(2÷3\right)\cdot 10^{-1}$ cm; α—is the angle of incidence of light on the site of the photosensor α=90°; β—is the angle between the normal to the reflective surface and the direction of the reflective side of the bio-tissue β=70°; r—the radius r=0.7 cm.

Assuming that $I_{X}=\frac{W}{\tau_{oпт}}$ the final conversion equation for the photodetector is written as follows [24,25]:

(3) $$I_{f}=-S_{I\lambda }\cdot \tau_{\lambda }\cdot I\cdot 10^{-3}\cdot \frac{\tau_{oпт}^{2}\cdot \pi \cdot r^{2}}{l^{2}\cdot L^{2}}\cdot \cos\alpha \cdot \cos\beta \cdot I_{x}.$$

From (3), analytical dependence for assessing the sensitivity of optical sensor is obtained

(4) $$S_{\mathrm{SO}}\frac{dI_{f}}{dI_{X}}=S_{I\lambda }\cdot \tau_{\lambda }\cdot I\cdot 10^{-3}\cdot \frac{\tau_{oпт}^{2}\cdot \pi \cdot r^{2}}{l^{2}\cdot L^{2}}\cdot \cos\alpha \cdot \cos\beta =const.$$

Considering (4), Equation (3) is given as:

(5) $$I_{f}=S_{SO}\cdot I_{X}.$$

**Figure 2:**
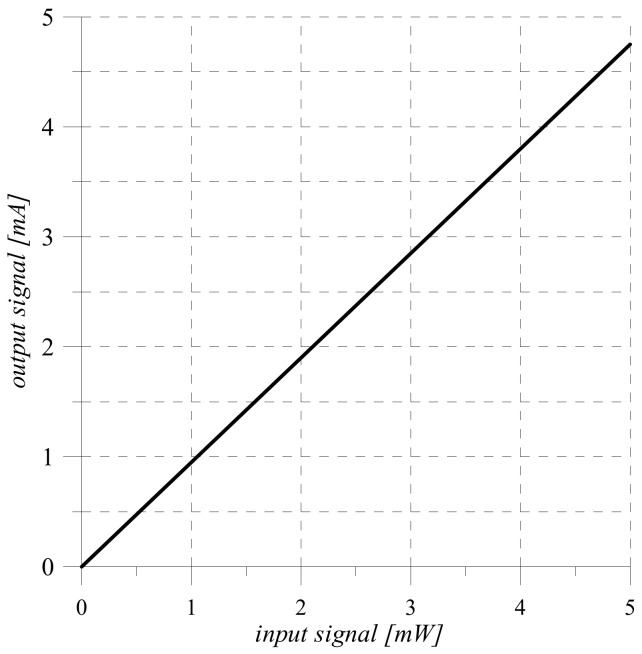
Static characteristic of the sensor.

Since the sensitivity of the sensor is a constant value, the obtained transformation Equation (4) and its static characteristic (Figure 2) are linear.

Using a shunt resistor, the current is converted into voltage, and this measurement transformation is mathematically represented as follows [24,25,26]:

(6) $$U=I_{f}\cdot R_{s}=\left(-S_{I\lambda }\cdot \tau_{\lambda }\cdot I\cdot 10^{-3}\cdot \frac{\tau_{opt}^{2}\cdot \pi \cdot r^{2}}{l^{2}\cdot L^{2}}\cdot \cos\alpha \cdot \cos\beta \cdot I_{x}\right)\cdot R_{s}.$$

**Figure 3:**
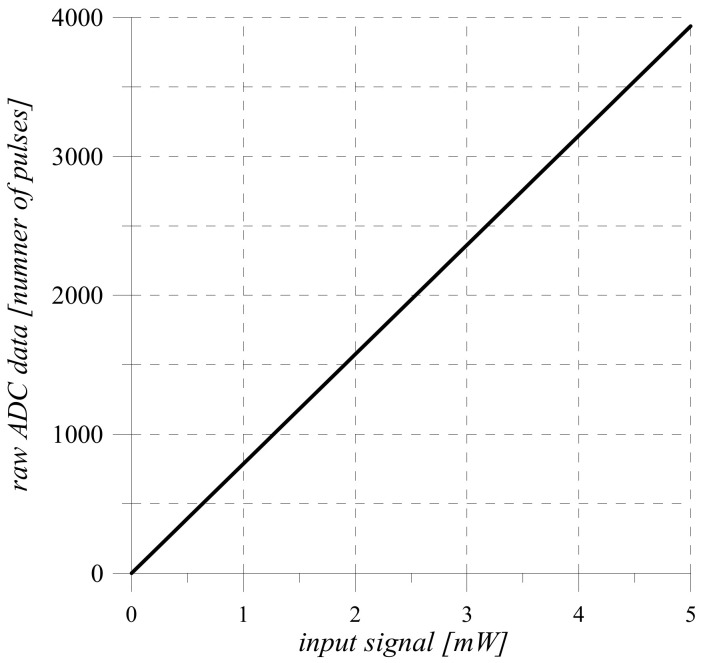
Voltage changing at the output of the shunt.

Since the range of voltage change (Figure 3) at the output of the shunt (*D* ∈ 0 ÷ 5 V) corresponds to the area of the possible change of the input signal at the analog input $I_{n}$ analog-to-digital converter ADC, the proposed scheme of the measuring channel (Figure 1) does not need scale transformation [27,28,29,30].

Thus, with the help of measuring devices (sensor and shunt) hardware and using the obtained conversion Functions (3) and (6) mathematically describes the process of converting a non-electric analog value of optical intensity $I_{X}$ into analog electric value—voltage *U*, which range is sufficient for reliable operation of the analog-to-digital converter [31,32].

## 3. Analog-to-Digital Conversion

Conversion of analog voltage *U* into digital binary code N is carried out as a result of analog-to-digital conversion, which is based on such methods of experimental computer science as reproduction, comparison, counting [33].

**Figure 4:**
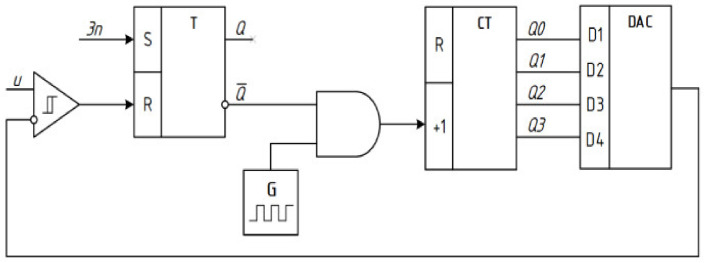
Block diagram of the sequential approximation ADC.

The block diagram for modeling the sequential approximation ADC is shown in Figure 4.

These methods [8] are realized by the following elements of the given diagram:

1. Counting—generator G, logic gate “AND”, n-bit binary counter CT;
2. Reproduction of the sample value (quantization step h) provides n-bit digital-to-analog converter DAC with an internal reference voltage source $U_{0}$; and
3. Comparison of the analog measured voltage *U* and the sample value *h* is performed by a comparator.

During the time of analog-to-digital conversion $t_{ADC}\;$logic gate “AND” is open and pulses with a frequency $f_{0}$ from the output of the generator G are fed to the input of the binary counter CT, counting their quantity N. From the output of the counter binary code is fed to the inputs of digital-to-analog converter DAC.

With the arrival of each frequency pulse $f_{0}$, the voltage value at the output of the DAC increases by the value of the quantization step [33,34,35]

(7) $$h=\frac{U_{0}}{2^{n}}\;.$$

The step-quantized voltage formed at the DAC output increases at the second input of the comparator until its value becomes equal to the value of the measured voltage *U* supplied to the first input of the comparator.

The main operation performed during the conversion of analog voltage into binary code is to compare the measured *U* and sample *h* values

(8) $$N=\frac{U}{h}.$$

Taking into account (8) and (6), the final equation of transformation of the measuring channel of optical intensity takes the form

(9) $$N=\frac{U}{h}=\frac{2^{n}}{U_{0}}\cdot U=\frac{2^{n}}{U_{0}}R_{s}\left(-S_{I\lambda }\cdot \tau_{\lambda }\cdot I\cdot 10^{-3}\cdot \frac{\tau_{opt}^{2}\cdot \pi \cdot r^{2}}{l^{2}\cdot L^{2}}\cdot \cos\alpha \cdot \cos\beta \right)\cdot I_{x}$$

and due to the sensitivity equation of the sensor (4) the transfer function of the measuring channel in analytical form is given as

(10) $$N=\frac{S_{SO}\;R_{s}\;2^{n}}{U_{0}}\cdot I_{X}$$

From the transfer Function (10) we obtain the sensitivity equation for a given measuring channel

(11) $$S_{BK}=\frac{d\;N}{d\;I_{X}}=\frac{S_{SO}\;R_{s}\;2^{n}}{U_{0}}=const\;.$$

**Figure 5:**
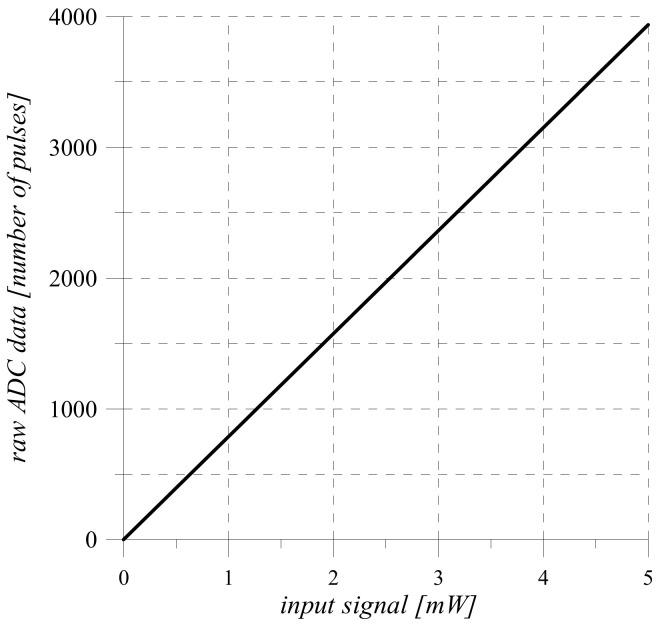
Static characteristics of the measuring channel.

Analysis of the sensitivity Equation (11) of the measuring channel shows that its transfer Function (10) and static characteristics (Figure 5) are linear.

## 4. Transmission and Evaluation of the Measuring Information Amount

Exporting the binary code N [00…07] from the ADC outputs to the RAM (Figure 1) can be implemented in different ways: by hardware or software, serial or parallel interface. Data can be transmitted in program mode, interrupt mode, and capture mode (direct access to memory) [36,37].

Data transmission does not convert measurement information, but the interface (parallel or serial) and transmission mode (software, interrupt, capture) significantly affect the performance of the measurement channel.

We will consider this statement in the example of using a parallel interface and software mode of information exchange between the outputs of the ADC and the MPS accumulator [38,39,40].

**Figure 6:**
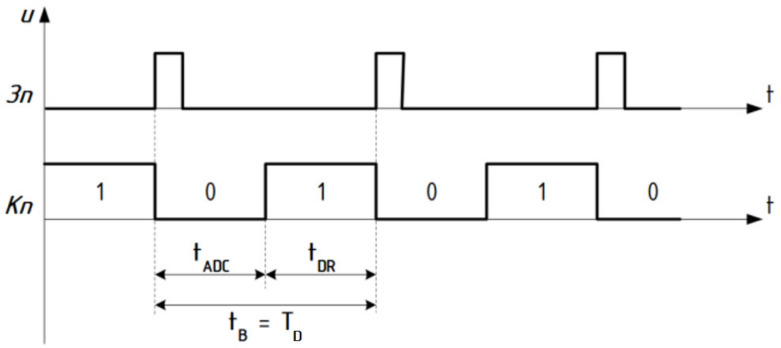
ADC timing diagrams.

According to signal *3n* (Figure 1 and Figure 6) the process of AD-conversion begins and at the output, the level of logical zero *Kn*: = “0” is formed.

The measurement process in this case is carried out by hardware and software. Hardware in the ADC implements a sequential approximation of the algorithm, which is performed during the time *t_ADC_*. The polling program is responsible for the software support of the measurement process. A flag register byte is introduced into the microprocessor system (MPS) accumulator from the PC port of the parallel PPI interface and its contents are checked for the presence of a logical unit “1” in one of the previously allocated bits, such as PC [02]. If the flag is missing *Kn*: = “0”, then the cycle of polling the flag is repeated until the process of AD-conversion is completed. If the flag *Kn*: = “1” is present, in the accumulator of the microprocessor system is writing a byte of information from the digital outputs of the ADC (or two bytes, if the bit ADC is greater than 8). The time that must be spent on the pling program operation is denoted by *t_DR_*.

Analysis of the above-mentioned time diagrams (Figure 6) shows that the speed *t_B_* of the measuring channel has the following two components

(12) $$t_{B}=t_{ADC}+t_{DR}.$$

The time *t_B_* required to obtain one measured value is the same that a sampling step

(13) $$T_{D}=t_{B}=t_{ADC}+t_{DR},$$

which is one of the components of dynamic error—sampling error:

(14) $$\Delta_{D}=\frac{1}{2}\;T_{D}\frac{d\;I_{X}}{d\;t}$$

The value of $t_{ADC}$ is determined by the time of analog-to-digital conversion, and its numerical value is obtained from the passport data of the ADC. To increase performance, it is necessary to choose an ADC with a minimum AD conversion time. To reduce the same component of the error, it is also necessary to reduce $\;t_{DR}$, which value is estimated by listing of the software driver. In the interrupt mode $\;t_{DR}$ less than in the program mode (by the value of the time spent on polling the flag—the readiness of the AC conversion). The highest data transfer speed provides direct access to memory, because there is no need for a software driver. Here the speed is determined only by the time on the AC transformation, since $\;t_{DR}$ = 0. Depending on the problem to be solved, the developer chooses an ADC with the ADC conversion time that is necessary to ensure the minimum value of sampling error $\Delta_{D}\to min$.

Determine the range of measurement of optical intensity (area of the input value change), in which the measuring instrument provides a given accuracy (normalized value of relative quantization error—accuracy class). The lower boundary of measurement scale $I_{X\;min}$ is limited by the normalized value $\delta_{KH}$ of relative quantization error.

(15) $$I_{X\;min}=\frac{U_{0}\;100\%}{\delta_{KH}\;S_{SO}\;R_{S}\;2^{n}}\;,$$

and the upper boundary r is limited by the maximum capacity $N_{max}=2^{n}$ of the binary counter CT2:

(16) $$I_{X\;max}=\frac{\;2^{n}\;h}{\;S_{SO}\;R_{S}}\;.$$

**Figure 7:**
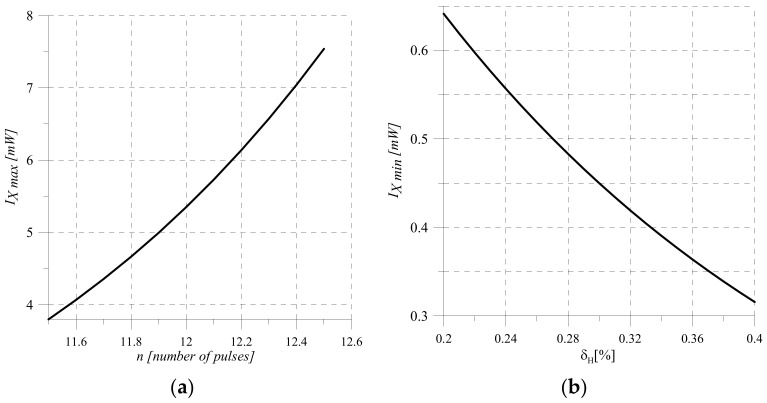
To the range of measuring transformation. (**a**) Dependence $I_{X\;min}=f\left(\delta_{KH}\right)$, (**b**) Dependence $I_{X\;max}=f\left(n\right)$.

Graphical dependences of the lower boundary of measurement $I_{X\;min}$ on the normalized value of the quantization error $\delta_{KH}$ and the upper boundary of measurement on the bit n of the binary counter ADC are shown in Figure 7.

As a result of the research, there was an opportunity to model the first obtained dependences (9)–(13), which will allow at the design stage of the measuring channel to obtain parameters for its structure that will convert the measured value into binary code in the range $I_{X\;min}$ to $I_{X\;max}$ with a predetermined normalized value of the relative quantization error [41,42,43].

Analysis of the sensitivity Equation (11) suggests that by choosing its components it is possible to achieve the value of the quantization error within $\delta_{KH}\in \left\{0.1\%–0.5\%\right\}$ In this range of possible changes in the quantization error, respectively, it is possible to perform (Figure 7) intensity measurements in the range from 1 to 100 mW.

The information obtained during the measurements [9] can be quantified by decreasing the entropy $H\left(I_{X}\right)$, which characterizes the uncertainty of the optical intensity before the measurement, to the value of $H\left(I_{X}/\delta_{KH}\right)$, which remains after obtaining the measurement result:

(17) $$I_{I}=H\left(I_{X}\right)-H\left(I_{X}/\delta_{KH}\right)$$

These estimates of uncertainty in the form of entropy before and after measurements are determined on the basis of *K*. Shannon’s 16th theorem, which is described by the functional [10,11,44]:

(18) $$H\left(I_{X}\right)=-\overset{+\infty }{\underset{-\infty }{\int }}P\left(I_{X}\right)\cdot log_{2}P\left(I_{X}\right)dI_{X}.$$

Measurements of optical intensity are carried out by a measuring channel (Figure 1), in which the measurement range is limited by the lower $I_{X\;min}$ and the upper $I_{X\;max}$ limits (Figure 7). Then the probability of obtaining the measurement result in the region of change $I_{X}$ from −∞ to $I_{X\;min}\;$and from $I_{X\;max}$ to +∞ is zero.

Therefore, the measurement result of the physical quantity $I_{X}$ must be expected in the measurement range from $I_{X\;min}$ to $I_{X\;max}$. Assume that the measurement result is equally likely to fall into any part of this range [45,46,47].

Therefore:

(19) $$P\left(I_{X}\right)=\frac{1}{I_{X\;max}-I_{X\;min}}\;\;\;\;for\;\;\;I_{X\;min}\leq \;I_{X}\;\leq \;I_{X\;max};$$

(20) $$P\left(I_{X}\right)=\;0\;\;\;\;\;for\;\;I_{X}\;<\;I_{X\;min}\;\;i\;\;I_{X}\;>\;I_{X\;max}.\;$$

taking into account the latter a priori determined entropy to measure

(21) $$H\left(I_{X}\right)=-\overset{+\infty }{\underset{-\infty }{\int }}\frac{1}{I_{X\;max}-I_{X\;min}}\cdot log_{2}\frac{1}{I_{X\;max}-I_{X\;min}}\;dI_{X}.$$

After performing the measurement, the entropy cannot be reduced to zero, because there is always uncertainty introduced by the error.

Determine the differential residual entropy for the case when the measurement error is distributed according to the normal law [12,48] and its standard deviation is equals σ

(22) $$P\left(I_{X}/\delta_{KH}\right)=\frac{1}{\sigma \;\sqrt{2\;\pi }}\cdot \exp\left(-\left({\left(\frac{\delta_{KH}}{2\;\sigma }\right)}^{2}\right)\right).$$

Then the posteriori entropy (after) the measurements is determined [8]

(23) $$H\left(I_{X}/\delta_{KH}\right)=-\overset{+\infty }{\underset{-\infty }{\int }}P\left(I_{X}/\delta_{KH}\right)\cdot log_{2}P\left(I_{X}/\delta_{KH}\right)\;d\;I_{X}\;log_{2}\left(\sigma \;\sqrt{2\;\pi \;e}\right).$$

The solution of the last equation is obtained by *K*. Shannon and has the following final form [47,49,50].

The amount of information obtained in the process of measuring the optical intensity of the proposed measuring channel is determined by the difference between a priori and posteriori entropy

(24) $$I_{I}=H\left(I_{X}\right)-H\left(I_{X}/\delta_{KH}\right)=-\overset{+\infty }{\underset{-\infty }{\int }}\cdot log_{2}\frac{1}{I_{X\;max}-I_{X\;min}}\;dI_{X}\;-log_{2}\left(\sigma \;\sqrt{2\;\pi \;e}\right).$$

For the set measuring range (Figure 7) and the standard deviation of the quantization error for (24), the amount of information of the measuring channel of optical intensity is $I_{I}=12\;$bits. This result does not contradict the empirical methods for determining the bit size of the analog-to-digital converter [40,47,49,50].

## 5. Requirements for Optical Luminous f51lux Sensors Example

The study of the pulse waves (FIG) parameters allows us to get a correct idea of a blood circulation parameters number. Arterial peripheral pulse is the result of the interaction of various oscillatory and wave processes. The volume pulse wave consists of two types of waves: connected with release and movement of systolic volume of blood and created by the hydraulic shock arising in a phase of the maximum expulsion of blood. In general, the shape of a volumetric pulse wave is determined mainly by the process of expulsion of blood from the ventricles of the heart and vibrations that occur both in the heart and in adjacent arterial vessels, as well as the damping effect of the vascular wall and surrounding organs and tissues.

The emitters must provide radiation in two spectral bands in the red and infrared ranges, the wavelength of radiation in the maximum of the spectral band (max) must be in the range of 650–670 nm for red glow and 800–1000 nm for infrared radiation, the radiation spectrum must be one of the narrowest possible band and no sidebands, high external quantum radiation output (VN) to obtain the maximum values of radiation power (Re) at low supply currents.

Linear dependence of radiation power on current in a wide range of currents.

High speed, which provides the ability to use emitters in different pulse modes.

Small single design with flexible tape leads for emitters on both spectral ranges or small design containing two crystals in one housing the distance between the emitting crystals in both cases should not exceed 3 mm.

For matrix processing of optical signals, optoelectronic signal converters must provide the required maximum sensitivity of 10–5 and 10–6 lux.

Ensure the homogeneity of the photodetector cells with a spread of not more than 2–5%.

All photodetectors as converters of optical signals in IIS are subject to the following requirements:

- Spectral distribution of photosensitivity, which corresponds to the spectral characteristics of the radiation source;
- High photosensitivity, which determines the minimum level of the input signal at a given level of output;
- Low noise level in a given frequency band and a given gain, which determines a low sensitivity threshold and high detection ability;
- Set electrical parameters: resistance, capacitance, voltage, and current, which determine the coordination of the photodetector with the load; and
- Wide bandwidth and large dynamic range for both optical input and electrical output, which leads to high speed and the possibility of analog conversion.

Different types of emitters are used in AF sensors. Depending on the sensitivity of the device, the radiation source is also selected. The use of low-power light emitters is not effective, as the photocell begins to act on the oscillations of extraneous light, and therefore for the sensor, it is necessary to create a case of opaque fabric, which disrupts the heat transfer of the studied organ. In addition, a further strong gain of the useful signal is required. At the same time, a powerful emitter causes the tissues to heat up.

The emitters must provide radiation in two spectral bands in the red and infrared ranges, the wavelength of the radiation in the maximum of the spectral band must be in the range of 650–670 nm for red glow and 800–1000 nm for infrared radiation, the radiation spectrum has one maximum narrow band, high external quantum radiation output, to obtain maximum values of radiation power at low supply currents, the linear dependence of radiation power on current in a wide range of currents, high speed, which allows the use of emitters in different pulse modes of operation, as well as low design with flexible tape leads for emitters on both spectral ranges or small design containing two crystals in one housing, the distance between the emitting crystals in both cases should not exceed 3 mm.

### 5.1. Requirements for Optical Sensors

One of the problems that arise in the development of photosensors in the AF is to ensure a large dynamic range (more than 150 dB) and high accuracy of signal measurement from the sensor. This follows from the need to measure the constant and variable (pulse) components that have passed through the tissues (or reflected) light, and their ratio may be at the level of tenths of a percent. In addition, the optical characteristics of the patient’s tissue have great variability.

Different types of photosensors can be used for FIGs: photomultipliers, photodiodes, photoresistors, valve photocells, emission type photocells. Each of these devices has its advantages and disadvantages. Extremely sensitive photoresistors are unstable with fluctuations in external temperature. In addition, their sensitivity in the infrared region was low. Photomultipliers are large and require a high voltage current source, which necessitates additional human protection. Photocells of the emission type are bulky, which prevents the examination of such parts of the body as the fingers, mucous membranes of the nose, throat, and others.

### 5.2. Requirements for Emitters

The emitters in both spectral ranges are made in a single small polymer housing with a size of 2.5 × 2.5 × 2.4 mm with deep tape leads.

Since the crystals used are transparent to the generating radiation and have significant lateral radiation, the holder contains a built-in light reflector, which allows us to increase the radiation power in each direction by 1.5–2 times.

To obtain an optimal radiation angle of 50 ± 10°, the housing has a hemispherical polymer dome with a radius R = 1 mm, which is located relative to the crystal in such a way that the S/R ratio is in the range of 1.6–1.7.

### 5.3. Main Parameters

1. Emitters with red and infrared radiation based on Ga_1−*x*_Al*_x_*As are characterized by a combination of high values of external quantum radiation output and speed. There are prospects for increasing *η_BH_* of infrared diodes to 30–35% and red LEDs to 15–20%.
2. Recently developed light diodes may also be of interest to the medical industry:
  - With a blue glow, on SiC-6H (*λ*_ma__x_—470–280 nm half-width Δ*λ*_1__/2_ = 60 nm) with *η_BH_* = 10–2%;
  - With a violet glow on SiC-4H (*λ*_ma__x_ = 423 nm, Δ*λ*_1__/2_ = 25 nm) with *η_BH_* = 10–3%; and
  - With a yellow glow with GaAs_0__.__15_ P_0__.__85_:N/CaP (*λ*_ma__x_ = 580 nm, Δ*λ*_1__/2_ = 30 nm) with *η_BH_* = 0.25% and many others.

One of the problems that arise in the development of optoelectronic analyzers of photoplethysmography signals is the provision of a large dynamic range (more than 150 dB) and high accuracy of signal measurement from a photo sensor. It follows from the need to measure the constant and variable (pulse) components that have passed through the fabric (or reflected) light, and their ratio may be at the level of tenths of a percent. In addition, the optical characteristics of the patient’s tissue have great variability. Thus, in the traditional ideology of the device there is a need to use 16-bit analog-to-digital converters (ADC) and preamplifiers with a programmable conversion ratio controlled, as a rule, by a microprocessor [30].

When recording processes in biological tissues, there is no need for a high sampling frequency of the signal, in particular, the spectrum of the photoplethysmogram is limited from above to 10–15 Hz. In this regard, it is proposed to use in this area sigma-delta-ADC with a high bit rate (up to 24) and with a working bandwidth of tens–hundreds of hertz.

### 5.4. Requirements for Luminous Flux Transformation Systems

When passing through the tissue, the radiation is scattered, getting on the skin located next to the photocell. Therefore, vascular reactions can be recorded at 10 mm away from the light source. The rays of light emanating from the illuminator must be collected in a single cylindrical beam equal in diameter to the photocell and directed to its working surface.

It is advisable to use a light guide, which gives, on the one hand, the ability to record vascular reactions with a certain size of the skin (usually only 1 cm^2^), and on the other, reduces the thermal effect of light on the tissue. In addition, it protects the photocell and the light emitter from sweat condensation.

The function of the optical fiber is also the supply of a parallel beam to the object of study. Quantitative determination of the distribution of blood supply using the Lambert–Beer formula is possible only for a parallel beam of light. To transmit light over a distance, special-shaped optical fibers are used, that give a complete internal reflection. When the working area of the photocell is small, the fiber is made in the form of a truncated cone. The working end can be given any shape that allows to conduct research on a certain area of skin, such as 1 cm^2^. The fiber with a variable cross-section is wide facing the light source, and narrow—the study object. This significantly increases the concentration of radiation in cases where a low-power light source is used.

During the development of the complex, several requirements were put forward to the created device:

- Availability of two channels for obtaining biomedical information; and
- Interaction and exchange of information with the computer via the interface.

**Figure 8:**
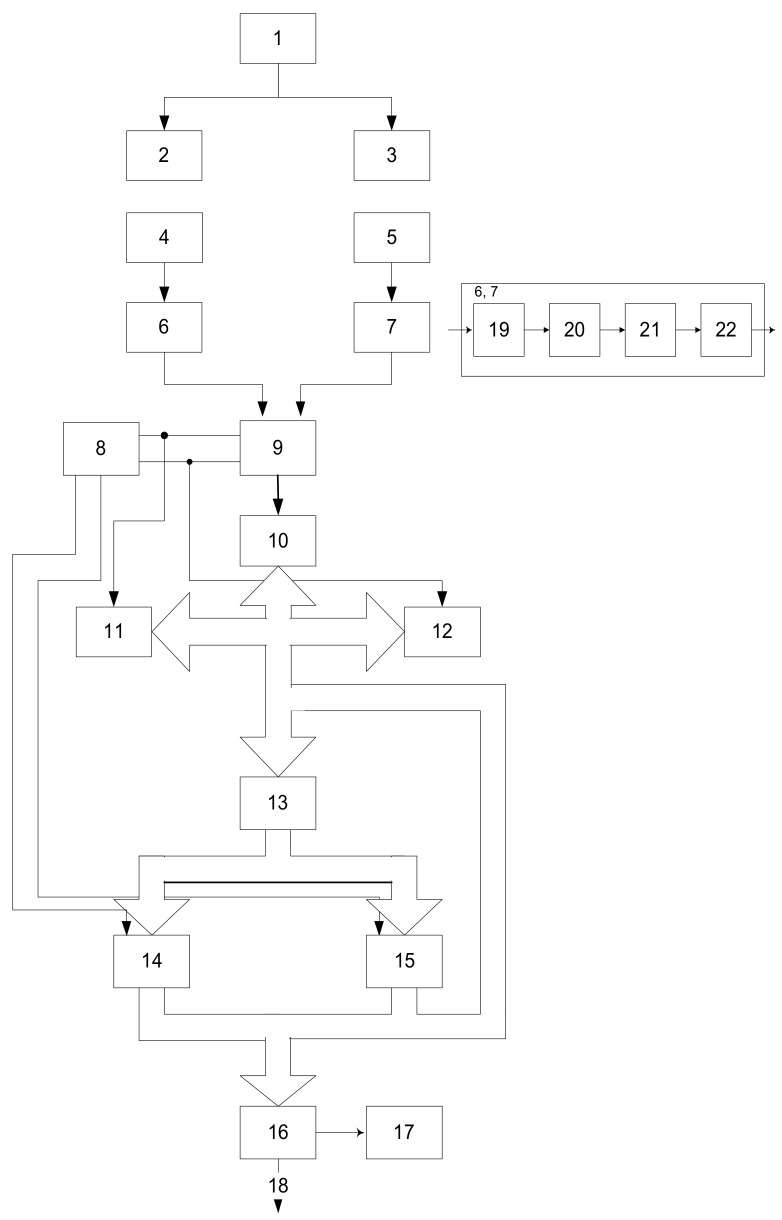
Block diagram of a device for diagnosing vascular disorders.

The device schematically shown in Figure 8 contains a source 1 of pulse voltage, the first 2 and the second 3 source of infrared radiation (LEDs), the first 4 and second 5 receivers of infrared radiation (photodiodes), the first 6 and second 7 blocks of analog signal processing, control unit 8, analog multiplexer 9, analog-to-digital converter (ADC) 10, first 11 and second 12 display units, register 13, first 14 and second 15 random access memory (RAM), microprocessor (MP) 16, display 17, information output of the device 18.

The output of the source 1 of the pulse voltage is connected to the inputs of the first 2 and second 3 sources of IR radiation, the optical outputs of which are connected respectively to the optical inputs of the first 4 and second 5 IR receivers, the outputs of which are connected to the inputs of the first 6 and the second 7 analog signal processing units, the outputs of which are connected to the input of the analog multiplexer 9, the address inputs of which are connected to the first and second inputs of the control device 8, and the output to the ADC input 10, the output bus of which is connected to the inputs of the first 11 and a second 12 display units, the control inputs of which are connected to the corresponding address inputs of the multiplexer 9 and to the input of the register 13, the output bus of which is connected to the inputs of the first 14, second 15 RAM, the control inputs of which are connected to the outputs, the output buses of the RAM are connected to each other and connected to the input of the register 13 and the MP 16, the output of which is connected to the output of the device 18 and the display 17.

The device works as follows. Paired photometric sensors consisting of sources 2, 3 of IR radiation and photodetectors 4, 5 are located in the intervertebral depressions symmetrically relative to the spine. The pulse voltage source 1 forms a periodic sequence of rectangular pulses that are fed to the sources 2, 3 of the IR radiation operating in the pulse mode. Pulses of radiation, passing through the studied vessels, are modulated by the amplitude of blood flow pulsations.

Modulated radiation flux is converted by photosensors 4, 6 into an electrical signal, which is pre-amplified by the amplifier 19. Next, the signal is amplified by the AC amplifier 20 at the operating frequency of the pulse voltage source 2. From the output of the demodulator 21, the signal 1 through the low-pass filter 22 having a bandwidth corresponding to the spectrum of the pulse wave signal is fed to the input of the analog multiplexer 9, through which the corresponding signals from the control unit 8 are fed to the ADC 9 in the digital code required for the operation of the MP 16. The control unit 8 controls the operation of the RAM, producing at the outputs of the decoder 25 signals to the inputs of the RAM recording permission.

## 6. Practical Implementation

Today, more and more methods, based on the use of optoelectronic devices, are being introduced into medical diagnostics. These include the photoplethysmographic method (PPM), which measures blood flow in strong veins and arteries, as well as in peripheral vessels and capillaries [31,32,33].

PPM, in comparison with other means of diagnostics of a biological object (BO) on optical indicators, for example, with a photoacoustic method, distinguishes by the simplicity of devices for its realization, and also that introduction in photoplethysmographic (PPG) devices of elements of light fiber technics and sources with different wavelengths of probing radiation, it is possible to easily solve the problems of photodynamic research, remote measurement of certain parameters of the desired BO, etc.

At this stage, the introduction of PPGs in medical practice PPG has not yet found its wide application for a number of reasons. One of them is the lack of biophysical justification for obtaining a photoplethysmographic signal.

There are two types of PPM—PPG in transmitted light and PPG in reflected light. Most often studies are performed in transmitted light, because in this case the direct assessment of blood supply in the required area of the BO is possible. But it is often quite difficult to conduct such research, for example, for optically opaque BO or for hard-to-reach areas of objects. Then use of the PPG method in reflected light, which not only allows to assess the total blood flow in the study area, but also gives an integrated assessment of the properties of the study surface.

In the case of PPG in reflected light, i.e., when the photoplethysmographic transducer (PMT) perceives the reflected radiant flux from the BO, it is shown that PPM allows to record the magnitude of changes in blood supply to the pulsation closest to PMT of the studied light flux depending on the amplitude of the pulsation of the tissue.

The usage of optoelectronic and laser sensors in biology and medicine can be carried out in several areas, one of which can be considered the development of new optoelectronic and laser technologies for the detection, identification, study of biological objects, as well as to study the nature of the processes, occurring in them.

**Figure 9:**
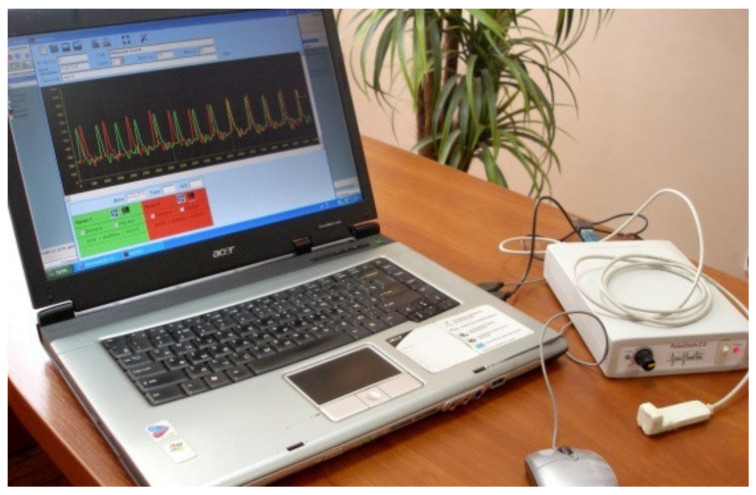
Optoelectronic device for diagnosing peripheral blood circulation.

**Figure 10:**
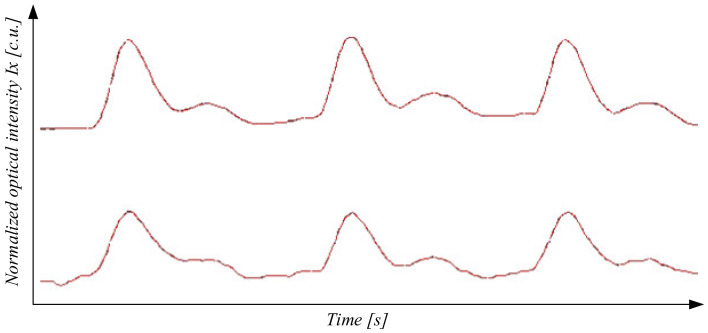
An example of deriving the results of the study.

Figure 9 shows the implementation of an optoelectronic sensor to determine peripheral blood flow and an example of the results of the study, shown in Figure 10.

### The Characteristics of the Optoelectronic Sensor

The emitters must provide radiation in two spectral bands in the red and infrared ranges, and the wavelength of the radiation in the maximum of the spectral band (max) must be in the range of 600–800 nm for red glow and 800–1000 nm for infrared radiation. To process optical signals, optical sensors must provide the required maximum sensitivity of 10–5 ÷ 10–6 lux. The following requirements apply to all photodetectors as optical signal converters: spectral distribution of photosensitivity, which corresponds to the spectral characteristics of the radiation source; high photosensitivity, which determines the minimum level of the input signal at a given level of output; low noise level in a given frequency band and a given gain, which determines a low sensitivity threshold and high detection capability; set electrical parameters: resistance, capacitance, voltage and current, which determine the coordination of the photodetector with the load; wide bandwidth and large dynamic range for both optical input and electrical output, which leads to high speed and the possibility of analog conversion.

When choosing photosensors for an optical sensor, there is a requirement to ensure a large dynamic range (more than 150 dB) and high accuracy of signal measurement from the sensor. This is due to the need to measure the constant and variable (pulse) components that reflected light, and their ratio may be at the level of tenths of a percent.

Red and IR wavelengths radiation output could be changed in the interval 90–280 µW, while the blue radiation output is constant. A new method was used for measuring PPG signals—digital PPG without analogue amplifier and filters. The signals acquired from measuring photodiode discharge time are inverse to the absorption of the light. The emission wavelengths were 660 nm and 880 nm.

For the task of registering a photoplethysmogram under the conditions of artifacts:

- A sensor with radiation sources evenly distributed around the circumference of the working surface, in the center of which a photodetector with a collecting lens is installed; and
- A sensor with an emitting surface formed using an optical system based on an LED, located in the center of the working surface of the sensor.

The authors of the article received more than ten patents of Ukraine for the implementation of an optical sensor and an optical-electronic device for the study of peripheral blood flow [29,30,32].

To increase the reliability of photoplethysmographic information, a priori information is used, which includes the physical characteristics of the object of study, mathematical relationships between the measured values, data on the spectral composition of informative components and interference, and basic biophysical characteristics of the controlled object.

## 7. Conclusions

On the example of the optical intensity measuring channel, the measurement result of which is given in binary code, the following phases of information conversion are highlighted: hardware implementation and mathematical description of the conversion of analog physical quantities in sensor and measuring current-to-voltage converter. shunt. The obtained conversion Functions (3) and (6) are the source for modeling the processes of conversion of analog information, the results of which are shown in Figure 2 and Figure 3.

Having applied the methods of experimental computer science of reproduction, comparison, and calculation on the example of ADC of sequential approximation the process of converting the analog value I_X into binary code N is described analytically, the function of converting a continuous value to a discontinuous value, sensitivity equation is obtained and measuring range is determined where the normalized value of the quantization error is provided for the measuring channels with optoelectronic sensors.

It is shown that the choice of the type of information transmission and interface in microprocessor measuring instruments significantly affects the performance of the measuring channel, which has two components: hardware and software. To establish the measurement range, based on the uncertainty in the form of entropy before and after the measurements, the amount of information the numerical value of which coincides with the empirical methods for determining the bit size of the analog-to-digital converter is estimated, which is also extremely important for for the developer at the design stage of digital measurement channels.

Analysis of the sensitivity equation suggests that by choosing its components it is possible to achieve the value of the quantization error within $\delta_{KH}\in \left\{0.1\%–0.5\%\right\}$. In this range of possible changes in the quantization error, respectively, it is possible to perform intensity measurements in the range from 1 to 100 mW.

The application of the results obtained in the work allows even at the design stage of physical and mathematical modeling to assess the basic static metrological characteristics of measuring channels, aimed at reducing the stage of development and debugging of hardware and software and standardization of their metrological characteristics.

## Data Availability

The data were taken from the scientific reports of the Faculty for Power Engineering and Electromechanics, Vinnytsia National Technical University, Laboratory of Biomedical Optics, Faculty for Infocommunications, Radioelectronics and Nanosystems, Vinnytsia National Technical University, Lublin University of Technology and Department of “Engineering and Technology” of the Azov Maritime Institute, NU “Odessa Maritime Academy”.

## References

[1] Kozhemyako V.P., Gotra Z.Y., Pavlov S.V. (2002). Circuitry of Modern Device Building: Part 3 Optical Sensors.

[2] Dorozhynsky G.V., Maslov V.P., Ushenin Y.V. (2016). Sensor Devices Based on Surface Plasmon Resonance.

[3] Kukharchuk V.V., Kucheruk V.Y., Volodarsky E.T., Grabko V.V. (2013). Fundamentals of Metrology and Electrical Measurements: A Textbook.

[4] Ornatsky P.P. (1994). Introduction to the Methodology of the Science of Measurement.

[5] Allen J. (2007). Photoplethysmography and its application in clinical physiological measurement. Physiol. Meas..

[6] Kozlovska T.I., Zlepko S.M., Kolesnic P.F. Optoelectronic multispectral device for determining the state of peripheral blood circulation. Proceedings of the Photonics Applications in Astronomy, Communications, Industry, and High Energy Physics Experiments 2020.

[7] Khairullina A.J. (1996). Multi wavelenght pulse oximetry in the measurement of gemoglobin fractions. Proceedings of the Photonics West 1996.

[8] Kukharchuk V.V., Vedmitsky Y.G., Volodarsky E.T. (2014). Function of analytical quantization of continuous, piecewise continuous and discrete signals. Metrol. Devices.

[9] Novitsky P.V., Zograf I.A. (1985). Measurements Results Errors Estimation.

[10] Shannon K. (1963). Mathematical Theory of Communication, Works on the Theory of Information and Cybernetics.

[11] Shannon K. (1963). Modern Achievements of Communication Theory, Works on Information Theory and Cybernetics.

[12] Malikov M.F. (1949). Fundamentals of Metrology, Part 1.

[13] Vasilenko A.M., Zhukolenko L.V., Ponnova A.M. (1998). Tensogalometry. Russ. Med. J..

[14] Goloshchapov-Aksenov R.S., Kurdo S.A., Sitanov A.C. (2013). Treatment of patients with critical ischemia of the lower extremities with common atherosclerotic lesion of the arterial bed. Int. J. Interv. Cardioangiol..

[15] Zaitseva T.A. (2014). Combined method of stimulation of neoangiogenesis in patients with critical ischemia of the lower extremities. Success Mod. Nat. Sci..

[16] Kaputin M.Y. (2008). Transluminal balloon angioplasty in patients with diabetes mellitus with critical ischemia of the lower extremities. Med. Acad. J..

[17] Kaputin M.Y. (2010). Balloon angioplasty in the treatment of critical ischemia of the lower extremities. Mod. Technol. Med..

[18] Kislov E.E. (2009). The results of treatment of patients with critical ischemia of the lower limb and distal arterial lesions. Ural Med. J..

[19] Ovcharenko D.V. (2011). Peripheral Angioplasty (PAP) with atypical blood supply in patients with critical ischemia. Int. J. Interv. Cardioangiol..

[20] Pityk A.I., Prasol V.A., Boyko V.V. (2014). Revascularization of the lower extremities in patients with critical ischemia due to infra-red blood vessel arteries. Angiol. Vasc. Surg..

[21] Sedov V. (2011). Effectiveness of cell therapy in patients with critical ischemia of the lower extremities. Reg. Blood Circ. Microcirc..

[22] Abdelhamid M.F. (2010). Below-the-ankle angioplasty is a feasible and effective intervention for critical leg ischaemia. Eur. J. Vasc. Endovasc. Surg..

[23] Brochado F.C. (2010). Inframalleolar bypass grafts for limb salvage. Eur. J. Vasc. Endovasc. Surg..

[24] Masaki H., Tabuchi A., Yunoki Y., Watanabe Y., Mimura D., Furukawa H., Yamasawa T., Honda T., Takiuchi H., Tanemoto K. (2014). Bypass vs. endovascular therapy of infrapopliteal lesions for critical limb ischemia. Ann. Vasc. Dis..

[25] Romiti M., Albers M., Brochado-Neto F.C., Durazzo A.E.S., Pereira C.A.D.B., De Luccia N. (2008). Meta-analysis of infrapopliteal angioplasty for chronic critical limb ischemia. J. Vasc. Surg..

[26] Soga Y., Iida O., Kawasaki D., Hirano K., Yamaoka T., Suzuki K. (2012). Impact of cilostazol on angiographic restenosis after balloon angioplasty for infrapopliteal artery disease in patients with critical limb ischemia. Eur. J. Vasc. Endovasc. Surg..

[27] Pavlov S.V., Kozlovska T.I., Vasilenko V.B. (2014). Opto-Electronic Devices for Diagnosis of Peripheral Circulation with High Reliability. Ph.D. Thesis.

[28] Pavlov S.V., Sander S.V., Kozlovska T.I., Kaminsky A.S., Wojcik W., Junisbekov M.S. (2013). Laser photoplethysmography in integrated evaluation of collateral circulation of lower extremities. Proceedings of the Optical Fibers and Their Applications 2012.

[29] Sander S.V., Kozlovska T.I., Vassilenko V., Pavlov V.S., Klapouschak A.Y., Kisała P., Romaniuk R., Sagymbekova A. (2015). Laser photoplethysmography in integrated evaluation of collateral circulation of lower extremities. Opt. Fibers Appl..

[30] Wójcik W., Smolarz A. (2017). Information Technology in Medical Diagnostics.

[31] Vassilenko V., Valtchev S., Teixeira J.P., Pavlov S. (2016). Energy harvesting: An interesting topic for education programs in engineering specialities. Internet Educ. Sci. IES.

[32] Pavlov S.V., Kozhemiako V.P., Kolesnik P.F. (2010). Physical Principles of Biomedical Optics.

[33] Pavlov S.V., Kozhemiako V.P., Petruk V.G., Kolesnik P.F. (2007). Photoplethysmohrafic Technologies of the Cardiovascular Control.

[34] Wójcik W., Pavlov S., Kalimoldayev M. (2019). Information Technology in Medical Diagnostics II.

[35] Pavlov S.V., Kozhukhar A.T., Titkov S.V., Barylo O.S., Sorochan O.M., Wójcik W., Romaniuk R., Zyska T., Annabaev A. (2017). Electro-optical system for the automated selection of dental implants according to their colour matching. Przegląd Elektrotechniczny.

[36] Kholin V.V., Chepurna O.M., Shton I.O., Voytsehovich V.S., Azarov O.D., Pavlov S.V., Gamaleia N.F., Harasim D. Methods and fiber optics spectrometry system for control of photosensitizer in tissue during photodynamic therapy. Proceedings of the Photonics Applications in Astronomy, Communications, Industry, and High-Energy Physics Experiments.

[37] Rovira R.H., Tuzhanskyy S.Y., Pavlov S.V., Savenkov S.N., Kolomiets I.S., Stasenko V.A., Bayas M.M., Omiotek Z., Małecka-Massalska T., Dzierżak R. Polarimetric characterisation of histological section of skin with pathological changes. Proceedings of the Photonics Applications in Astronomy, Communications, Industry, and High-Energy Physics Experiments.

[38] Zabolotna N.I., Pavlov S.V., Radchenko K.O., Stasenko V.A., Wójcik W. (2015). Diagnostic efficiency of Mueller-matrix polarization reconstruction system of the phase structure of liver tissue. Proceedings of the Optical Fibers and Their Applications 2015.

[39] Avrunin O.G., Tymkovych M.Y., Pavlov S.V., Timchik S.V., Kisała P., Orakbaev Y. (2015). Classification of CT-brain slices based on local histograms. Optical Fibers and Their Applications 2015.

[40] Kukharchuk V.V., Hraniak V.F., Vedmitskyi Y.G., Bogachuk V.V., Zyska T., Komada P., Sadikova G. Noncontact method of temperature measurement based on the phenomenon of the luminophor temperature decreasing. Proceedings of the Photonics Applications in Astronomy, Communications, Industry, and High-Energy Physics Experiments.

[41] Chepurna O., Shton I., Kholin V., Voytsehovich V., Popov V., Pavlov S., Gamaleia N., Wójcik W., Zhassandykyzy M. (2015). Photodynamic therapy with laser scanning mode of tumor irradiation. Proceedings of the 16th Conference on Optical Fibers and Their Applications.

[42] Dubolazov A.V., Koval G.D., Zabolotna N.I., Pavlov S.V. Fractal structure of optical anisotropy Mueller-matrices images of biological layers. Proceedings of the Eleventh International Conference on Correlation Optics.

[43] Zabolotna N.I., Wojcik W., Pavlov S.V., Ushenko O.G., Suleimenov B. (2013). Diagnostics of pathologically changed birefringent networks by means of phase Mueller matrix tomography. Proceedings of the Optical Fibers and Their Applications 2012.

[44] Rovira J.R., Pavlov S.V., Vassilenko V.B., Wójcik W., Sugurova L. (2013). Methods and resources for imaging polarimetry. Proceedings of the Optical Fibers and Their Applications 2012.

[45] Zabolotna N.I., Pavlov S.V., Ushenko A.G., Karachevtsev A.O., Savich V.O., Sobko O.V., Olar O. System of the phase tomography of optically anisotropic polycrystalline films of biological fluids. Proceedings of the SPIE NanoScience + Engineering.

[46] Zabolotna N.I., Pavlov S.V., Ushenko A.G., Sobko O.V., Savich V.O. (2014). Multivariate system of polarization tomography of biological crystals birefringence networks. Biosensing Nanomed..

[47] Vedmitskyi Y.G., Kukharchuk V.V., Hraniak V.F. (2017). New non-system physical quantities for vibration monitoring of transient processes at hydropower facilities, integral vibratory accelerations. Przegląd Elektrotechniczny.

[48] Tymchenko L., Tverdomed V., Petrovsky N., Kokryatskaya N., Maistrenko Y. (2019). Development of a method of processing images of laser beam bands with the use of parallelhierarchic networks. East. Eur. J. Enterp. Technol..

[49] Kukharchuk V.V., Kazyv S.S., Bykovsky S.A. (2017). Discrete wavelet transformation in spectral analysis of vibration processes at hydropower units. Przegląd Elektrotechniczny.

[50] Kukharchuk V.V., Bogachuk V.V., Hraniak V.F., Wójcik W., Suleimenov B., Karnakova G. Method of magneto-elastic control of mechanic rigidity in assemblies of hydropower units. Proceedings of the Photonics Applications in Astronomy, Communications, Industry, and High Energy Physics Experiments.

